# Mitochondrial disease-related mutations at the cytochrome *b*-iron–sulfur protein (ISP) interface: Molecular effects on the large-scale motion of ISP and superoxide generation studied in *Rhodobacter capsulatus* cytochrome *bc*_1_^☆^

**DOI:** 10.1016/j.bbabio.2016.03.022

**Published:** 2016-08

**Authors:** Robert Ekiert, Arkadiusz Borek, Patryk Kuleta, Justyna Czernek, Artur Osyczka

**Affiliations:** Department of Molecular Biophysics, Faculty of Biochemistry, Biophysics and Biotechnology, Jagiellonian University, 30-387 Kraków, Poland

**Keywords:** ETC, electron transport chain, ISP, iron–sulfur protein, ISP-HD, head domain of ISP, WT, wild type, DBH_2_, 2,3-dimethoxy-5-methyl-6-decyl-1,4-benzohydroquinone, SOD, superoxide dismutase, EPR, electron paramagnetic resonance, CW, continuous wave, ROS, reactive oxygen species, Cytochrome *bc*_1_, Mitochondrial complex III, Mitochondrial diseases, Reactive oxygen species, Electron transfer, Domain movement

## Abstract

One of the important elements of operation of cytochrome *bc*_1_ (mitochondrial respiratory complex III) is a large scale movement of the head domain of iron–sulfur protein (ISP-HD), which connects the quinol oxidation site (Q_o_) located within the cytochrome *b*, with the outermost heme *c*_1_ of cytochrome *c*_1_. Several mitochondrial disease-related mutations in cytochrome *b* are located at the cytochrome *b-*ISP-HD interface, thus their molecular effects can be associated with altered motion of ISP-HD. Using purple bacterial model, we recently showed that one of such mutations — G167P shifts the equilibrium position of ISP-HD towards positions remote from the Q_o_ site as compared to the native enzyme [Borek et al., J. Biol. Chem. 290 (2015) 23781-23792]. This resulted in the enhanced propensity of the mutant to generate reactive oxygen species (ROS) which was explained on the basis of the model evoking “semireverse” electron transfer from heme *b*_L_ to quinone. Here we examine another mutation from that group — G332D (G290D in human), finding that it also shifts the equilibrium position of ISP-HD in the same direction, however displays less of the enhancement in ROS production. We provide spectroscopic indication that G332D might affect the electrostatics of interaction between cytochrome *b* and ISP-HD. This effect, in light of the measured enzymatic activities and electron transfer rates, appears to be less severe than structural distortion caused by proline in G167P mutant. Comparative analysis of the effects of G332D and G167P confirms a general prediction that mutations located at the cytochrome *b*-ISP-HD interface influence the motion of ISP-HD and indicates that “pushing” ISP-HD away from the Q_o_ site is the most likely outcome of this influence. It can also be predicted that an increase in ROS production associated with the “pushing” effect is quite sensitive to overall severity of this change with more active mutants being generally more protected against elevated ROS.

This article is part of a Special Issue entitled ‘EBEC 2016: 19th European Bioenergetics Conference, Riva del Garda, Italy, July 2–6, 2016’, edited by Prof. Paolo Bernardi.

## Highlights

• Several mitochondrial mutations are located at the cytochrome *b-*ISP interface.
• We compare molecular effects of two mutations from that group.
• In both mutants ISP is shifted away from the Q_o_ catalytic site.
• This effect is generally associated with increased ROS production.
• More active mutants are more protected against elevated ROS.

## 1 Introduction

Cytochrome *bc*_1_ (mitochondrial complex III), a part of electron transport chain (ETC), is involved in building of proton motive force which is utilized to produce ATP [1]. This protein is a homodimer, in which each monomer contains from 3 to 11 subunits, depending on species [2]. The catalytic core of this protein consists of only 3 subunits: cytochrome *b*, cytochrome *c*_1_ and iron–sulfur protein (ISP). Its function is related to electron transfer from quinol to cytochrome *c* and the translocation of protons across the membrane. Communication between these two pools of electron carriers is important for energetic efficiency of ETC [3].

In the catalytic Q cycle of cytochrome *bc*_1_ [4], [5], a complete cycle of reactions occurs in two chains of cofactors embedded in the catalytic core. In the high-potential c-chain, quinol oxidation site (Q_o_ site) is connected with cytochrome *c* reduction site by two cofactors: Rieske cluster and heme *c*_1_, embedded in ISP and cytochrome *c*_1_, respectively. Movement of the head domain of iron–sulfur protein (ISP-HD) between a site close to the Q_o_ site (Q_o_ position) and a site on the cytochrome *c*_1_ interface (c_1_ position) allows transfer of electrons via this chain [6], [7], [8], [9], [10], [11]. In the low-potential b-chain the Q_o_ site is connected with quinone reduction site (Q_i_ site) by heme *b*_L_ and heme *b*_H_ embedded in cytochrome *b*. Completion of the Q cycle requires two quinol molecules to be oxidized at the Q_o_ site. After oxidation of first molecule of quinol at the Q_o_ site one electron is transferred via high-potential c-chain and is used to reduce one molecule of cytochrome *c*. The second electron derived from oxidation of first molecule of quinol is transferred via low-potential b-chain and is used to reduce quinone to semiquinone at the Q_i_ site. Electrons derived from oxidation of second quinol molecule are used to reduce one molecule of cytochrome *c* at the cytochrome *c* reduction site and to reduce semiquinone to quinol at the Q_i_ site. In addition, a cross-dimer electron transfer at the level of two hemes *b*_L_ is possible [12], [13].

Each monomer of the human cytochrome *bc*_1_ complex is composed of 11 subunits [2]. Among all of them, only cytochrome *b* is encoded by mitochondrial DNA, whereas all the other subunits are of nuclear origin. Numerous cytochrome *b* mutations identified so far are related with diseases such as exercise intolerance, miopathy, cardiomiopathy and neuropathies [14], [15], [16], [17]. These mutations mostly exhibit a deficiency in the enzymatic activity and decreased amount of some of complex III subunits. Some mutations may also cause an increase in superoxide radical production [18]. To study the molecular effects of human mitochondrial disease-related cytochrome *b* mutations, bacterial [19], [20] or yeast [17], [21] systems are used.

**Fig. 1:**
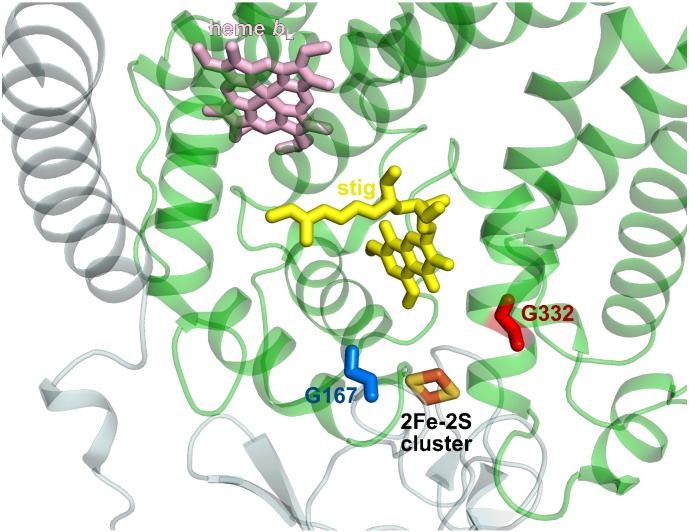
Position of Gly-332 in the structure of *R. capsulatus* cytochrome *bc*_1_. Close-up view of the crystal structure of cytochrome *bc*_1_ (PDB: 1ZRT[46]) showing part of cytochrome *b* (green) and ISP (light blue). Light pink and light orange sticks indicate heme *b*_L_ and [2Fe–2S] cluster, respectively. Yellow sticks represent the stigmatellin molecule in the Q_o_ catalytic site. Positions Gly-332 and Gly-167 are marked as red and blue sticks, respectively.

In the present study we have used the *Rhodobacter capsulatus* bacterial model to examine the molecular effects of mitochondrial mutation G290D, which was identified in patient suffering from progressive exercise intolerance [22]. Earlier study of mitochondria isolated from this patient's muscle showed a deficiency in the enzymatic activity of cytochrome *bc*_1_ and decreased amounts of some subunits: cytochrome *b* and ISP among others, while amounts of cytochrome *c*_1_ were normal [23], [24], [25]. Gly-290 position in cytochrome *b* is highly conserved among the species. It is localised in the transmembrane helix F in the proximity of the Q_o_ catalytic site but also in close contact with ISP-HD at the Q_o_ position (Fig. 1). The mutation in *R. capsulatus* equivalent to human G290D is G332D. The change from glycine to aspartic acid introduces structural change (bulkier side chain) and additional negative charge. It thus might perturb the structure of the Q_o_ site pocket and/or affect the interaction of ISP-HD with cytochrome *b*, which could influence the overall motion of ISP-HD. Recently, we found that another mitochondrial disease-related mutation G167P, located as G332D at the cytochrome *b*-ISP-HD interface, did affect the motion of ISP-HD which, among other effects, led to increased levels of superoxide production at the Q_o_ site [19]. In light of these findings we considered here G332D as a good candidate for the comparative analysis with G167P.

## 2 Materials and methods

### 2.1 Mutagenesis

*R. capsulatus* containing the substitution of glycine to aspartate in position 332 of cytochrome *b* subunit were obtained as follows. Mutation was introduced in the *petB* gene (coding for cytochrome *b*) using the QuikChange site-directed mutagenesis system (Stratagene) and the following PCR primers (the changed nucleotide in the underlined triplet is in bold): G332D_F: 5′-GATGCGAAGTTCTTCG**A**CGTGATCGCGATGTTCGGCGC-3′ and G332D_R: 5′-GCCGAACATCGCGATCACG**T**CGAAGAACTTCGCATCGAC-3′. As a template DNA pPET1 plasmid containing wild type (WT) *petABC* operon and a Strep-tag at the C-terminal end of cytochrome *b* was used [26]. The *BstX*I-*Asu*II fragment of the operon bearing the desired mutations was inserted into the pMTS1 vector and introduced into MT-RBC1 *R. capsulatus* strain using triparental crossing [27]. The presence of introduced mutation was confirmed by sequencing the whole *petABC* operon.

### 2.2 Bacterial growth

*R. capsulatus* cells were cultured in mineral-peptone-yeast-extract (MPYE) at 30 °C either semiaerobically (at low oxygen concentration, in dark) or photoheterotrophically (without oxygen, using BD GasPak system, in the presence of light) as described previously [26]. The growth rate estimations were performed as follows — a single colony of analysed strain and of apparently similar size was cultured semiaerobically in 2 ml of MPYE supplemented with 10 μg/ml kanamycin for ~ 14 h. 10 or 100 μl of such bacterial suspension was spread on MPYE/Kan agar plates and the growth under photosynthetic conditions was documented after 3, 5 and 7 days. 10 colonies of cells grown under photosynthetic conditions were re-streaked on new plates and their genotypes were tested by DNA sequencing. All cells contained the G332D mutation and no other mutations in the genes coding for all subunits of the *bc*_1_ complex.

### 2.3 Isolation of chromatophores and protein purification

Chromatophores were obtained from *R. capsulatus* cells grown under semiaerobic conditions as described previously [28]. After isolation, chromatophores were homogenized and suspended in MOPS buffer pH 7.0 containing 100 mM KCl and 1 mM EDTA. Chromatophores were solubilized with n-dodecyl-β-D-maltopyranoside detergent (DDM, Anatrace) for 45 min on ice (4 °C) and subsequently ultracentrifuged. Supernatant was used for purification of the cytochrome *bc*_1_ complexes using the Strep-tag affinity chromatography following the manufacturer's protocol (IBA) with modifications [26].

### 2.4 Steady-state kinetics and superoxide measurements

Enzymatic activities of isolated cytochrome *bc*_1_ complexes were determined spectroscopically under steady-state conditions, at the beginning of the 2,3-dimethoxy-5-methyl-6-decyl-1,4-benzohydroquinone (DBH_2_)-dependent reduction of cytochrome *c* (bovine heart cytochrome *c* obtained from Sigma-Aldrich) as described previously [27]. All enzymatic assays were performed in 50 mM Tris buffer pH 8.0, containing 0.01% DDM and 100 mM NaCl. Turnover rates in Table 1 were calculated from the initial linear parts of the time-dependent cytochrome *c* reduction kinetics. The kinetic parameters V_max_ and K_M_ were calculated by fitting Michaelis–Menten equation to data from Fig. 3. The superoxide radical production was determined from the difference between enzymatic activity with and without superoxide dismutase (SOD) and was shown in Fig. 7A as percentage above the bars [29], [30], [31]. The concentration of CuZn-SOD was 100 U/ml. Cytochrome *bc*_1_ complexes used in enzymatic assays were at final concentration of 10–100 nM. The final concentrations of cytochrome *c*, quinone pool (DBH_2_ + DB) and antimycin were 20 μM except for determination of V_max_ and K_M_ values where DBH_2_ concentration was in the range of 0.5 to 20 μM.

### 2.5 Light-induced electron transfer measurements

Double beam time-resolved spectrophotometer was used to measure the kinetic properties of cytochrome *bc*_1_ heme cofactors. Transient kinetics of hemes *b* (560–570 nm) and hemes *c* (550–540 nm) were obtained for chromatophores suspended in MOPS buffer pH 7.0 containing 100 mM KCl and 1 mM EDTA. The measurements were carried out under anaerobic conditions in the presence of 3.5 μM valinomycin and redox mediators as described in [32], [33]. Redox reactions were induced in chromatophores by single saturating light flash (50 ms, wavelength above 580 nm) and measured under conditions of pH 7.0 and 100 mV of ambient potential. The rates of flash-induced heme *b* reduction in the presence of antimycin (Sigma-Aldrich) were calculated using a single exponential equation fitted to kinetic transients.

### 2.6 Electron paramagnetic resonance (EPR) measurements

Continuous wave EPR spectra of [2Fe–2S] cluster were measured at X-band frequency (9.6 GHz) on chromatophores. Samples were prepared in MOPS buffer pH 7.0 containing 100 mM KCl and 1 mM EDTA. All spectra were measured at 20 K, using a SHQEOS11 resonator and the following parameters: resonance frequency, 9.39 GHz; microwave power, 1.545 mW; modulation amplitude, 15 G.

Pulsed EPR spectroscopy was used to obtain the temperature dependence of phase relaxation rates of the reduced [2Fe–2S] cluster as described previously [19], [34]. Measurements were performed at Q-band frequency (33.5 GHz) on isolated cytochrome *bc*_1_ complexes. Samples were prepared in Bicine buffer pH 8.0 containing 100 mM NaCl and 20% glycerol in the presence of reductant (1 mM sodium ascorbate) and measured in the temperature range of 12–23 K. All EPR measurements were carried on a Bruker Elexsys E580 spectrometer.

## 3 Results

### 3.1 G332D mutation affects the bc_1_-dependent growth of *R. capsulatus*

**Fig. 2:**
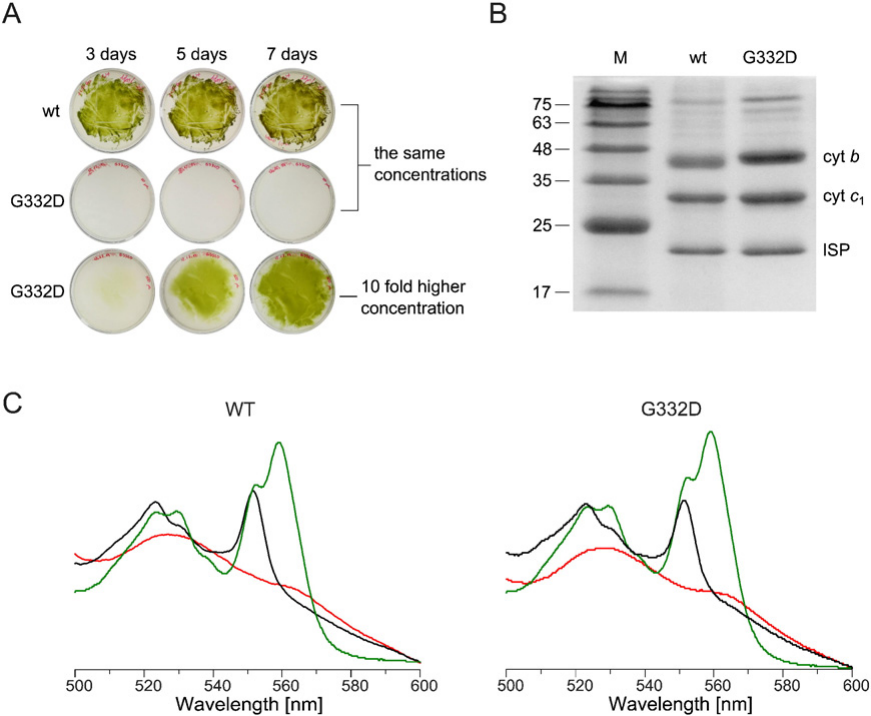
Photosynthetic growth, subunit composition and optical spectra of hemes in WT and G332D mutant. A – comparison of photoheterotrophic growth of wild type *R. capsulatus* and the G332D mutant after 3, 5 and 7 days of culture. To be able to observe the growth of the mutant cells they had to be seeded in about 10-fold higher concentration of bacteria than the wild type. B – SDS-PAGE analysis of the affinity-purified complexes; M – the protein mass marker in kDa. C – comparison of the optical spectra of hemes *b* and *c* of purified wild type and G332D complexes. Samples were oxidized with ferricyanide (red traces) and reduced with sodium ascorbate (black traces) or dithionite (green traces).

One of the ways to test the functionality of mutated cytochrome *bc*_1_ in bacteria is to observe its ability to sustain growth under the photosynthetic conditions. As the anoxygenic growth of *R. capsulatus* is dependent on the functional electron transport chain, the lower growth rate of bacteria bearing mutated cytochrome *bc*_1_ complex suggests impediments in the complex operation. Upon introducing the G332D mutation in the cytochrome *b* in *R. capsulatus* we observed that the growth rate of mutated bacteria was dramatically slower than in the wild type (Fig. 2A). When both strains were seeded in the same amount, the growth of G332D could not be detected even after 7 days of culture. However, when the mutated bacteria were seeded at 10-fold higher concentration, some growth was observed (Fig. 2A). Under aerobic conditions, independent of functional *bc*_1_, the G332D bacteria exhibited growth rate comparable with the wild type (data not shown).

### 3.2 G332D mutant shows native-like subunit composition and optical spectra of hemes

To test whether the G332D mutation affects the subunit composition of the *bc*_1_ complex we purified the complex using affinity chromatography. Visualization of the purified proteins on the SDS gel revealed that in *R. capsulatus* the mutated complex consists of all three catalytic subunits — cytochrome *b*, cytochrome *c*_1_ and ISP (Fig. 2B). We have also investigated the spectral properties of purified *bc*_1_ complexes bearing the G332D mutation in comparison to the native enzyme. Thus we measured optical absorption spectra of these proteins under various oxidative and reducing conditions (Fig. 2C). The spectra of oxidized hemes *b* and hemes *c*, obtained in the presence of 1 mM sodium ferricyanide (red traces) did not differ between G332D and WT. Addition of sodium ascorbate reduced heme *c* in G332D (appearance of absorption peak at 551 nm (Fig. 2C, black traces)) indicating that this heme is high potential as in the native enzyme. Addition of potassium dithionite resulted in both WT and G332D in the additional maximum of absorption at 560 nm (Fig. 2C, green traces) corresponding to low-potential hemes *b*. Moreover, the peak-to-peak ratio on dithionite-reduced spectra of G332D (551 nm: 560 nm) did not reveal any significant changes in comparison to WT indicating that the ratio of hemes *c* to *b* in the mutant *bc*_1_ complex was not changed.

### 3.3 G332D mutant exhibits lower enzymatic activity than wild type bc_1_ complex

**Fig. 3:**
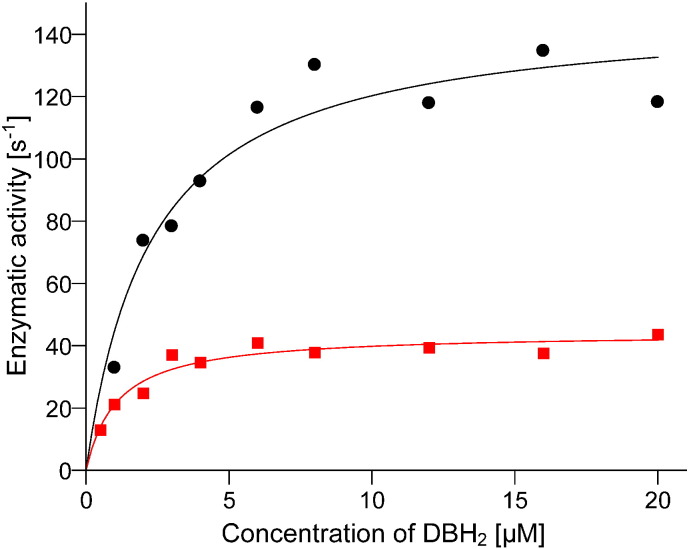
Comparison of enzymatic activities of WT and G332D mutant. The plots show dependence of the turnover rate of WT (black) and G332D mutant (red) in the function of quinol (DBH_2_) concentration. The Michaelis–Menten equation was fitted to the data points and yielded the V_max_ and K_M_ values.

The measurements of enzymatic activity at 20 μM cytochrome *c* and 20 μM quinol revealed that isolated *bc*_1_ complex bearing G332D mutation is approximately 3-fold less active than the wild type (Table 1). The same result of decrease enzymatic activity was obtained by measuring V_max_ for different concentrations of quinol with 20 μM cytochrome *c* (Fig. 3). When compared to the WT, G332D mutant exhibited 3-fold decline in V_max_ (45 ± 2 s^− 1^ and 146 ± 9 s^− 1^ for G332D and WT, respectively) and 2-fold decline in K_M_ (1.1 ± 0.2 μM and 2.3 ± 0.5 μM for G332D and WT, respectively).

**Fig. 4:**
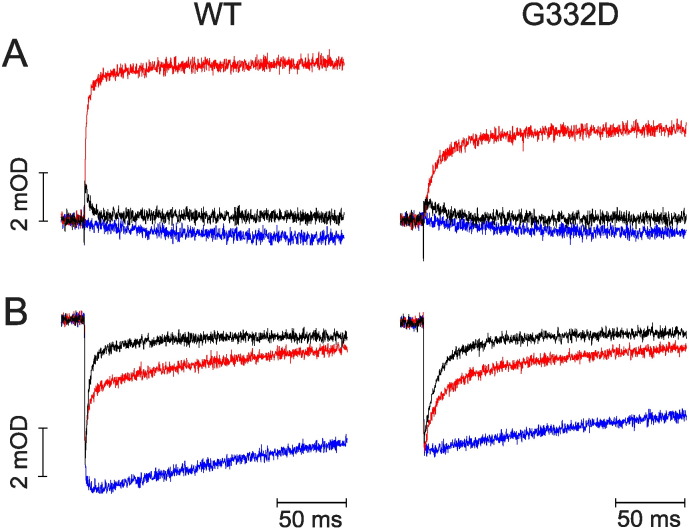
Comparison of light-activated kinetics of WT and G332D mutant. A – kinetic transients of hemes *b* reduction recorded at 560–570 nm, B – oxidation and re-reduction of hemes *c* observed at 550–540 nm. All traces were recorded at pH 7 and ambient potential of 100 mV without inhibitors (black), in the presence of antimycin (red) and antimycin with subsequent addition of myxothiazol (blue).

Using flash-induced double wavelength spectrophotometry we were able to monitor the redox reactions of heme cofactors within the *bc*_1_ complex in chrompatophores. Kinetic transients showed in Fig. 4 represent oxidation and reduction of hemes as a result of light-activated electron transfer through cofactor chains during the Q cycle. Transients of hemes *b* (Fig. 4A) obtained for G332D chromatophores in the presence of antimycin (red traces) indicate lower reduction rate in comparison to wild type (approximately 6-fold, Table 1). Also the rates of cytochrome *c* re-reduction are significantly slower for G332D mutant compared to WT both for non-inhibited (Fig. 4B, black traces) and antimycin-inhibited samples (Fig. 4B, red traces).

### 3.4 G332D mutation changes CW EPR spectrum of [2Fe–2S] cluster and affects the equilibrium position of ISP-HD

**Fig. 5:**
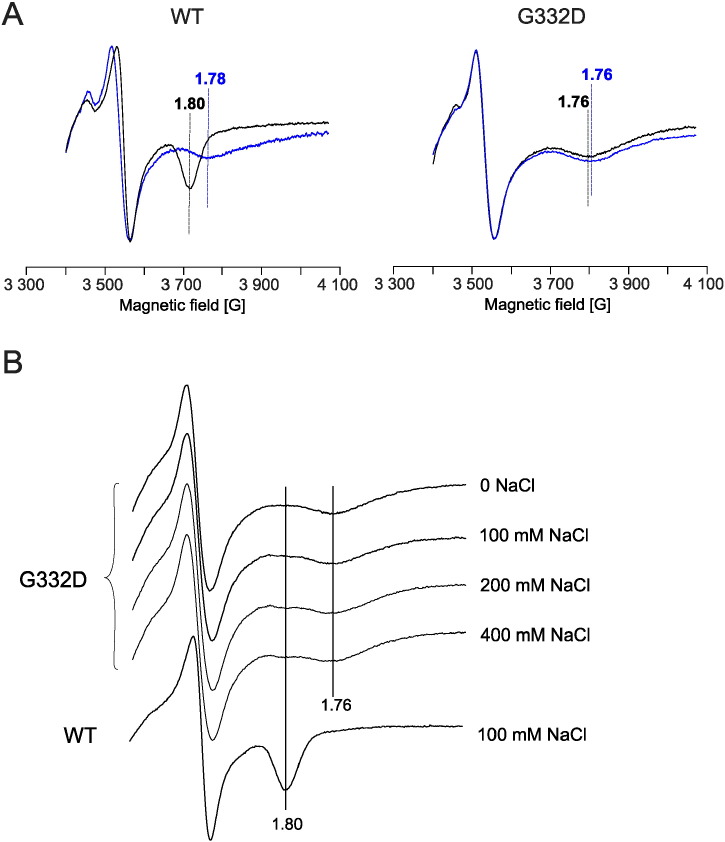
Comparison of EPR spectra of [2Fe–2S] cluster in WT and G332D mutant. A – X-band CW EPR spectra of [2Fe–2S] cluster of *bc*_1_ complex in chromatophores isolated from wild type and G332D mutant. Traces were recorded in the absence of inhibitors (black traces) or in the presence of myxothiazol (blue traces). B – change in shape of the EPR spectrum of [2Fe–2S] cluster in G332D in various salt concentrations.

Fig. 5 compares continuous wave electron paramagnetic resonance (CW EPR) spectra of WT and G332D mutant. Clearly, the characteristic resonance transition g_x_ = 1.80, reflecting the interactions of the reduced Rieske cluster with quinone in the Q_o_ site of the wild type complex [35], [36] was not observed in the G332D mutant (Fig. 5A). The spectrum of non-inhibited G332D mutant exhibited a g_x_ transition of 1.76, which is similar to a spectrum of WT after quinone extraction (empty Q_o_ site) [37]. Moreover, addition of myxothiazol did not alter the shape of G332D spectrum.

To investigate whether the presence of G332D influences the movement of the ISP-HD, which could be one of the reasons for the observed changes in the CW EPR spectra of [2Fe–2S] cluster in this mutant, we measured the temperature-dependence of phase relaxation rates of the cluster in wild type and in G332D cytochrome *bc*_1_. These measurements monitor the distance-dependent interactions between the [2Fe–2S] cluster and heme *b*_L_. The oxidized heme *b*_L_ can enhance the relaxation of reduced Rieske cluster in such a way that the closer these two paramagnetic centres are, the stronger the enhancement of phase relaxation is. We have successfully used this approach to estimate changes in the average position of [2Fe–2S] cluster in relation to other cofactors in the native enzyme and various mutants of *R. capsulatus* cytochrome *bc*_1_ [19], [31], [34].

**Fig. 6:**
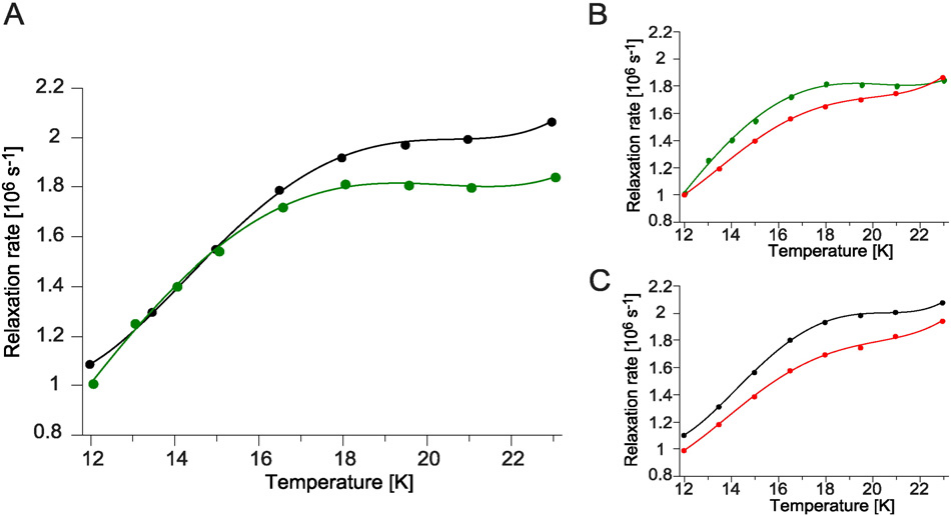
Temperature dependence of phase relaxation rate of [2Fe–2S] cluster in WT and G332D cytochrome *bc*_1_. Relaxation rates of the reduced [2Fe–2S] cluster were obtained by fitting one exponent to the electron spin echo decay measured by pulsed EPR at Q-band on isolated complexes as a function of temperature; A – wild type (black circles) and G332D (green circles). B – G332D without inhibitors (green circles) and antimycin-inhibited (red circles). C – WT without inhibitors (black circles) and antimycin-inhibited (red circles). All samples were reduced with sodium ascorbate.

Comparison of the relaxation rate profiles of non-inhibited wild type and G332D in Fig. 6A revealed weaker enhancement in the mutant. This indicates that the average equilibrium position of [2Fe–2S] cluster is shifted in G332D towards a *c*_1_ position, more remote from heme *b*_L_. In the presence of antimycin, G332D exhibited further decrease in relaxation rate (Fig. 6B) reminiscent of an even larger shift in the average position of the cluster away from the Q_o_ site. Similar structural effect was observed in the antimycin–inhibited wild type (Fig. 6C), as recognized earlier by this method [34] and in the EPR studies on oriented chromatophore membranes [38].

As the additional charge on the surface of cytochrome *b* in G332D mutant can possibly affect electrostatics of interaction between cytochrome *b* and ISP-HD, we measured CW EPR spectra of ISP in chromatophores in buffers with different concentration of NaCl. Interestingly, the increase of ionic strength resulted in the slight, but clearly distinguishable appearance of g_x_ = 1.80 transition at the expense of g_x_ = 1.76 transition (Fig. 5B). For the highest NaCl concentration (400 mM) the contribution of cytochrome *bc*_1_ complexes exhibiting the g_x_ = 1.80 transition (characteristic for native-like interaction of ISP-HD with the Q_o_ site) could be estimated for 2–4% of the whole population.

### 3.5 G332D mutation results in increased reactive oxygen species production in the presence of antimycin

**Fig. 7:**
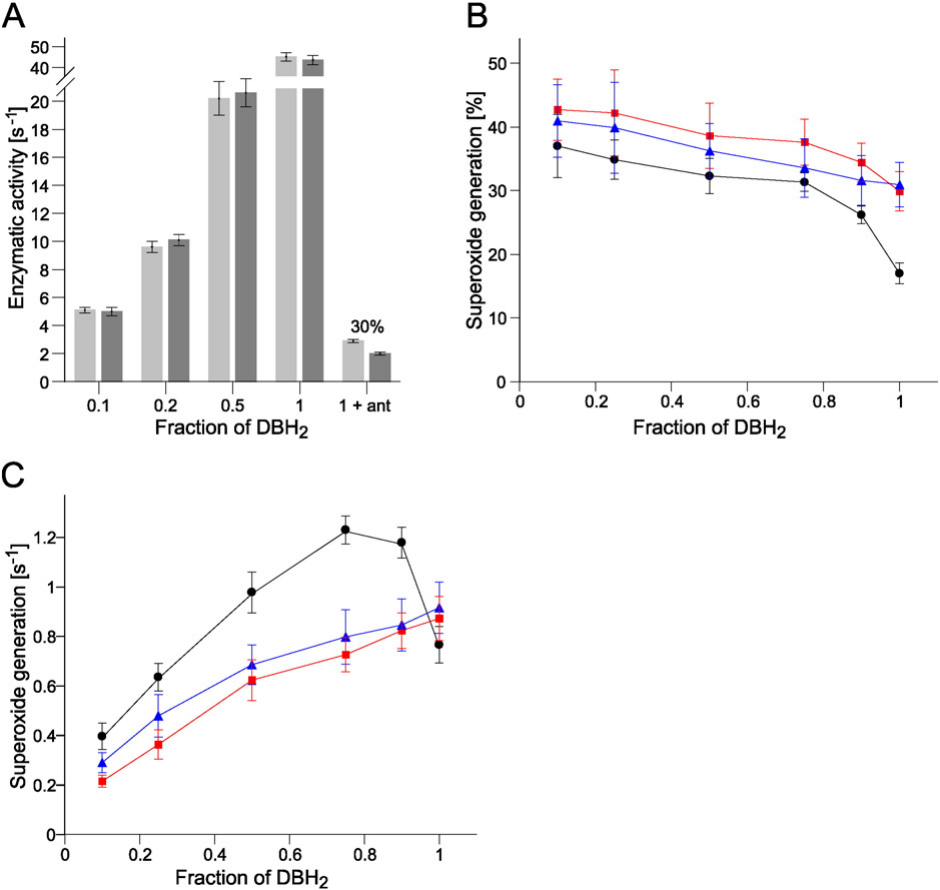
Comparison of enzymatic activities and ROS production. A – activities were measured at pH 8.0 and 100 mM NaCl under varying redox states of the Q pool (decreasing DBH_2_ to DBH_2_ + DB ratio) in the absence (light grey bars) and presence of SOD (dark grey bars). The last two bars represent enzymatic activities of antimycin-inhibited G332D in a fully-reduced Q pool. Superoxide radical production is shown as percentage above the bars. B – production of superoxide in antimycin-inhibited enzymes under varying redox states of the Q pool expressed “relatively” (as percentage of superoxide generated per single turnover). The total concentration of the Q pool was 20 μM. WT (black), G332D (red) and G167P (blue). C – “quantitative” production of superoxide (expressed as a number of μmol of superoxide produced by 1 μmol of the enzyme per second) corresponding to the plot shown in B. Error bars in A, B and C represent standard deviation of the mean of at least 4 measurements.

The increased distance between Rieske cluster and the Q_o_ site is one of the conditions promoting generation of superoxide by cytochrome *bc*_1_ [19], [30], [31]. Moreover, ROS production is influenced by the redox state of the quinone pool (the Q pool) exhibiting the characteristic bell-shaped dependence [19], [39], [40]. In the absence of any inhibitors, we could not detect any ROS in G332D mutant by the SOD-dependent measurements of superoxide production under variety of redox conditions: when the redox state of the Q pool became more oxidized (by decreasing the ratio of quinol to Q pool) the enzymatic activity of G332D cytochrome *bc*_1_ decreased to the same levels, regardless of the SOD presence (Fig. 7A). However, after addition of antimycin the superoxide production was detected at level exceeding that typically observed in WT under the same conditions ([19], see also Fig. 7B). We thus compared the dependence of ROS generation in various redox states of the Q pool in antimycin-inhibited G332D and antimycin-inhibited WT.

With increasing oxidation of the Q pool the amount of superoxide generation described “relatively” as a percentage of superoxide generated per single turnover increased for WT and G332D mutant (Fig. 7B). The difference in percentage of superoxide production between mutant and WT was more pronounced with the Q pool totally reduced (fraction of DBH_2_ = 1). Under this condition, G332D mutant revealed approximately 2-fold higher level of antimycin-dependent ROS generation than the WT (30% vs 17%, Fig. 7B). In antimycin-inhibited WT, the dependence of superoxide generation rate (described “quantitatively” as a number of μmol of superoxide produced by 1 μmol of the enzyme per second) was bell-shaped with the maximum ROS generation at a quinol to Q pool ratio of approximately 0.75 (Fig. 7C, black), consistent with previous observations [39], [40]. However, the G332D mutant did not show such bell-shaped dependence (Fig. 7C, red). This observation prompted us to examine antimycin-inhibited G167P, the mutant which without antimycin exhibited WT-like bell-shaped dependence [19]. Antimycin-inhibited G167P lacked the bell-shaped dependence (Fig. 7C, blue), similarly to antimycin-inhibited G332D.

## 4 Discussion

### 4.1 Comparison of structural effects associated with ROS production in G332D and G167P

Our results reveal that mutation G332D in cytochrome *b* of *R. capsulatus* influences the interaction of ISP-HD with cytochrome *b* rather than the affinity of cytochrome *bc*_1_ for quinol (a low K_M_ for G332D mutant indicates a high affinity of the enzyme for quinol (Fig. 3)). Based on EPR analysis of the CW and pulse EPR spectra of [2Fe–2S] cluster we concluded that this influence manifests itself in a shift in the equilibrium position of ISP-HD towards the position more remote from the Q_o_ site (in comparison to the native enzyme). This macroscopic effect appears similar to the shift observed earlier in G167P, another mutation located at the cytochrome *b*-ISP-HD interface. However, while in G167P this shift was associated with the ability of mutant to generate superoxide even in the absence of antimycin [19], in G332D generation of superoxide could only be observed in the presence of this inhibitor. This marks the major difference between these two mutants.

The increased propensity of cytochrome *bc*_1_ to generate superoxide in G167P [19] (in native complex no superoxide production is typically detected in the absence of antimycin) was explained on the basis of “semireverse” electron transfer favouring reactive oxygen species production [30], [31]. According to this mechanism, the back electron transfer from the reduced heme *b*_L_ to quinone results in formation of semiquinone at the Q_o_ site, which has the best chance to react with oxygen when the ISP-HD occupies positions remote from the Q_o_ site at the time of semiquinone formation. We thus predicted that mutations that shift the equilibrium position of ISP-HD towards a position more remote from the Q_o_ site should increase the level of ROS production. Indeed, both G167P and G332D appear to fall into this category of mutants showing elevated levels of ROS in the presence of antimycin (in comparison to wild type [19], [30]). However, the effect of G332D on ROS production is clearly more subtle comparing to G167P, because the former does not produce superoxide without inhibitors (Fig. 7A). To explain such difference one can propose that a shift in the average ISP-HD position is larger in G167P than in G332D. We note that the sensitivity of the method based on measurements of enhancement of [2Fe–2S] cluster relaxation decreases for larger distances between heme *b*_L_ and the cluster. This means that for the studied cases even if small changes in the average position between the mutants G167P and G332D exist, they may not necessarily be resolved by this method. However, the assumption that the shift in ISP-HD position may indeed be somewhat larger in G167P seems consistent with the observation that G332D did not exert as severe impediments for enzymatic/electron transfer activity (Table 1, Fig. 4) and photosynthetic growth (Fig. 2A) as G167P [19]. It is also reasonable from the structural point of view: insertion of proline in G167P may introduce more severe structural distortions than insertion of aspartic acid in G332D which, as visualized by the results of Fig. 5B, could predominantly affect the electrostatic interactions between cytochrome *b* and ISP-HD. It is tempting to speculate that the shift in G332D is at the borderline of structural change that just begins to result in elevated ROS production (seen experimentally only in the presence of antimycin) while the shift in G167P crosses that line and elevated ROS become detectable even in the absence of this inhibitor. It follows that within the group of enzymes shifting the average position of ISP-HD away from the Q_o_ site, even small difference in the shift (falling beyond the limits of detection by the described above enhancement method) may result in significant difference in the ROS generation at the Q_o_ site.

### 4.2 Effect of the redox state of Q pool on ROS production by G332D and G167P

Superoxide radical produced by cytochrome *bc*_1_ is a product of reaction of semiquinone generated at the Q_o_ site with oxygen. There are two possible ways, in which semiquinone can be created at this site: as a part of forward reaction, when first electron is transferred from quinol to the Rieskie cluster and the second electron transfer from semiquinone to heme *b*_L_ does not take place (“semiforward” reaction) [29], [41] or as a part of reverse reaction occurring when electron is transferred from heme *b*_L_ to quinone and the second electron transfer from Rieske cluster to semiquinone does not take place (“semireverse” reaction) [30], [31], [39], [42]. Both of these mechanisms can, in principle, explain the production of superoxide by the enzyme exposed to excess of quinol when oxidation of hemes *b* is impeded in the presence of antimycin.

These two mechanisms differ in terms of the substrate used to generate oxygen-reactive semiquinone. In the “semiforward” mechanism, the substrate is quinol while in the “semireverse” mechanism — it is quinone. This distinction guided the explanation of the previously observed shape of dependence of ROS generation rate in antimycin-inhibited submitochondrial particles on the redox state of the Q pool [39]. Because the maximum rate of superoxide generation was detected when the Q pool was partly oxidized, the characteristic bell-shaped curve was ascribed as indicative of occurrence of “semireverse” reaction. Recent studies revealed that such bell-shaped dependence can also be observed in the absence of antimycin. This concerned the mutant G167P, which unlike WT and G332D, generates superoxide even in the absence of this inhibitor [19].

Interestingly, such bell-shaped dependence was not observed in antimycin-inhibited G332D mutant. Moreover, antimycin-inhibited G167P mutant did not show this dependence either (Fig. 7C). To explain this observation one should consider that the presence of quinone at the Q_o_ site can have a dual effect on superoxide generation rate by cytochrome *bc*_1_. On one hand, quinone as a competitive inhibitor of the site decreases enzymatic activity of cytochrome *bc*_1_ (the major influence on activity is when Q pool is nearly or fully oxidized) and thus may reduce the rate of superoxide production calculated “quantitatively”. On the other hand, according to “semireverse” electron transfer, quinone as a substrate for semiquinone generated at the Q_o_ site increases the level of superoxide radical production calculated “relatively”. The competition between these two opposite effects impacts on the dependence of superoxide production rate on the fraction of DBH_2_. The lack of bell shape in both mutants in “quantitative” plot in the range between 0.5 and 1 (Fig. 7C) results from the lack of clear increment in levels of ROS in “relative” plot in the range between 0.75 and 1 (Fig. 7B). In WT, the increment concerns a change from 17% to nearly 40% of cytochrome *c* reduction rate while in the mutants it starts from the level as high as 30% and can further be increased only slightly, up to 43% which is close to theoretical limit of ROS generation (50%). Thus “quantitative” ROS production in WT can reach a maximum when the fraction of DBH_2_ is approximately 0.75 (this value is consistent with earlier observations reported for antimycin-inhibited submitochondrial particles [39] and non-inhibited G167P mutant [19]). For the antimycin-inhibited mutants, however, “quantitative” ROS production cannot increase significantly, as it would lead to levels of 50% or even more, which would mean a total disruption of the Q cycle.

Based on these results we anticipate that not all mutants of cytochrome *bc*_1_ will exhibit the characteristic bell-shaped dependence of ROS production on the redox state of Q pool, but only those in which the level of superoxide production in fully reduced state of quinone pool is at moderate levels (not exceeding 30%).

### 4.3 Comparison of G332D in *R. capsulatus* with other species

Similarly to our results, G332D in another species of photosynthetic purple bacteria, *Rhodobacter sphaeroides* also caused impediment in photosynthetic growth and decrease in enzymatic activity [43]. The EPR spectra of Rieske cluster in this mutant exhibit the g_x_ transition of 1.77 [44], but no further studies on this mutation were reported for this species. Mitochondrial complexes III from yeast and human bearing equivalents of this mutation (G291D and G290D, respectively) were enzymatically less active [21], [23], [24], [25]. Additionally, the yeast G291D mutant was unable to grow on the complex III-dependent respiratory medium. In both cases the authors ascribed these effects to the observed lower amounts of cytochrome *b* and ISP. It thus appears that mitochondrial complex III with this mutation is generally less stable. While this feature was not observed with bacterial cytochrome *bc*_1_, the decreased stability of complex III may be associated with the change in ISP-HD position observed in bacteria. Indication that such correlation is possible comes from the studies showing that the presence of antimycin (which, as G332D, shifts the ISP-HD away from the Q_o_ site) makes the complex more prone to the proteolytic cleavage of ISP-HD [45], while mutations and inhibitors acting in the opposite direction (i.e. fixing the ISP-HD at the Q_o_ site) protect the complex from such cleavage [11], [45]. We note that yeast mutant S152P (equivalent of *R. capsulatus* G167P mutation) showed almost complete lack of ISP subunit [21], which again seems to correlate with the shift in the ISP-HD away from the Q_o_ site observed in bacteria [19]. Based on these observations one can speculate that the correlation between molecular effect on the motion of ISP-HD (seen in G332D and G167P) and protein stability (seen in G291D and S152P) extends to other mitochondrial mutations in cytochrome *b*.

Interestingly, it was found that the respiratory dysfunction of G291D mutant in yeast was partially restored by the additional mutation D287H [21]. These two mutations are in the same region of cytochrome *b*, only 3 Å apart. It thus would seem that a change from acidic amino acid (aspartate) to positively charged histidine neutralizes the negative charge introduced at the position 291 and such a compensatory effect may be responsible for the partial restoration of the proper function of the complex. This observation further supports the idea of the electrostatic nature of the ISP-HD shift caused by the G332D mutation.

### 4.4 Conclusions

If one considers G332D and G167P as two representatives of the mutations that target the cytochrome *b*-ISP-HD interface exerting various structural distortions and/or changes in the surface charge (electrostatic potential) it can be generalized that these types of mutations influence the motion of ISP-HD with a shift in the average position of ISP-HD away from the Q_o_ site being the most likely outcome of this influence. This leads to an increase in ROS production consistent with the model evoking “semireverse” electron transfer from heme *b*_L_ to quinone as initial reaction leading to formation of semiquinone that can react with oxygen. The level of increase in ROS production and conditions under which ROS are observed can vary depending on severity of the structural change, with more active mutants (exhibiting larger enzymatic activity and electron transfer rates) being generally more protected against elevated ROS. To observe the characteristic bell-shaped dependence of ROS production on the redox state of the Q pool the tested form of cytochrome *bc*_1_ should display moderate levels of ROS under fully reduced state of the Q pool.

## Transparency document

Transparency document. Image 1

## Figures and Tables

**Table 1:** Enzymatic activity of wild type, G332D and G167P cytochrome *bc*_1_ complexes.

Strain	Enzymatic activity [1/s]^a^	Flash-induced heme *b* reduction [1/s]
WT^b^	140 ± 5	818
G332D	45 ± 2	140
G167P^b^	14.7 ± 0.2	16.6

a Errors represent standard deviation of the mean of 5–12 measurements.

b Data from [19].
